# Exploring Service Users’ Experiences of a Community-Based Intervention to Improve Follow-Up at Bharatpur Eye Hospital in Nepal: Qualitative Study

**DOI:** 10.2196/65023

**Published:** 2025-06-25

**Authors:** Manisha Shrestha, Gopal Bhandari, Sadhan Bhandari, Gudlavalleti Venkata Satyanarayana Murthy, Ruchi Priya, Binod Pandey, Daya Shankar Chaudhary, Puspa Giri, Sureshkumar Kamalakannan, Operational Research Capacity Building Study Group, Gudlavalleti Venkata Satyanarayana Murthy

**Affiliations:** 1 Bharatpur Eye Hospital, Bharatpur, Nepal; 2 Indian Institute of Public Health, Hyderabad, India; 3 Pragyaan Sustainable Health Outcomes Foundation, Hyderabad, India; 4 London School of Hygiene and Tropical Medicine, London, United Kingdom; 5 Department of Social Work Education and Community Well-Being, Northumbria University, Coach Lane Campus, Newcastle Upon Tyne, NE7 7TR, United Kingdom, 44 7366962444

**Keywords:** qualitative study, barriers, facilitators, Nepal, follow-up, eye care services, child health, follow-up care

## Abstract

**Background:**

Follow-up to eye care services for children, especially in the context of Nepal, is essential for ensuring a continuum of care. Hence, as a continued effort, we designed this study to explore the service users’ experience of a community-based intervention to improve follow-up at Bharatpur Eye Hospital (BEH) in Nepal.

**Objective:**

This study aimed to explore service users’ experiences and perceptions of the community-based follow-up intervention for eye care services implemented by BEH in Nepal.

**Methods:**

A qualitative study using an in-depth interview approach was used. Participants were purposively selected for this research study. Participants who were part of a quasi-experimental study conducted to improve follow-up services in BEH and their service catchment area were chosen. Participants who had not attended even a single follow-up visit and participants who attended at least one follow-up were recruited for this qualitative study. Based on the distance from the base hospital and the follow-up status, 65 participants were initially found eligible for the qualitative study. However, only 17 participants were available and consented. Topic guides were developed for the purpose of in-depth interviews specifically for participants who had not attended even the first follow-up visits and those who attended at least one follow-up visit. A total of 2 ophthalmic assistants, who were not a part of the main intervention study, conducted the interviews.

**Results:**

In total, 17 service users whose children were receiving services from BEH participated in the qualitative study. We identified 4 key themes that provided invaluable information about the barriers and facilitators to follow-up as well as the experiences (positive or negative) of the participants that need to be considered in any future initiatives to improve follow-up in Nepal.

**Conclusions:**

This study highlights the need for systematic development of interventions to address the unmet need for eye care services in the community through innovative, scalable solutions. As a next step, the BEH team will be working to develop such scalable solutions for Nepal. Such interventions will also need to be optimized for similar settings and countries to meet the goals of universal health coverage, vision 2030, and sustainable development goals worldwide.

## Introduction

### Background

Nepal’s National Health Policy 2019 recommends the development and expansion of eye care services through public-private partnerships in all 3 tiers of primary care provided through the government health systems. It provides guidance on the integration of primary eye care with primary health care and the coordination of eye care programs by a dedicated eye unit at the federal ministry of health [1]. One such effort is the Retina Eye Care of Nepal (RECON) project, which was designed to strengthen retina eye care services in Nepal [2]. This initiative was a well-planned health education intervention targeting physicians’ knowledge about diabetic retinopathy. Evidence from the evaluation of this initiative identified an increase in the number of referrals and attendance of patients for diabetic retinopathy screening with the change in knowledge and referral mechanism [3].

However, providing referrals does not mean the care is complete. Follow-up for eye care services for children, especially in the context of Nepal, is essential for ensuring a continuum of care [4]. Counseling and follow-up with patients are the key factors in improving the usage of health care services [5]. An exploratory analysis of data from one single hospital (Bharatpur Eye Hospital [BEH]), spanning from January 1, 2019, to January 7, 2019, revealed that only 22% of children aged 0-16 years attended at least one follow-up visit and the remaining 78% could not attend for various reasons. There are several studies conducted to find the impact of interventions, such as SMS text messaging, phone calls, and counseling to improve adherence to follow-up care in both adult and pediatric populations. One of the studies has shown that SMS text messaging reminders significantly improved follow-up adherence in pediatric cataract treatment compared to the control group [6].

A quasi-experimental study was carried out in Nepal by the BEH team to find the effectiveness of follow-up interventions, particularly counseling with the provision of information leaflets and reminders through phone calls and text messages to improve the follow-up rates in comparison to the existing standard care. The primary outcome of the study was measured by the number of children completing all 3 follow-up visits in different intervention groups. This research showed no statistically significant difference in the proportion of follow-up between the intervention groups. Only 3% (8/264) of children completed all 3 follow-ups, but the overall compliance with follow-up was only 0.76%, with more follow-ups in the reminder with SMS text messaging and phone call groups [7]. There may be several reasons for the poor adherence to follow-up visits, but it was hypothesized that COVID-19 was the major contributor since the study was conducted during the peak COVID-19 time. Hence, as a continued effort, we designed this study to explore the service users’ experience of a community-based intervention to improve follow-up at BEH in Nepal. We believed that this study would provide invaluable information on the reasons for the poor follow-up outcomes from the quasi-experimental study and the potential drivers of developing a culturally relevant, context-specific intervention for improving follow-up to ensure a continuum of eye care in the communities of Nepal.

### Primary Objective

The primary objective of this research study was to explore service users’ experiences and perceptions of the community-based follow-up intervention for eye care services implemented by BEH in Nepal.

## Methods

### Study Design

The study design was a qualitative study using an in-depth interview approach.

### Setting

The interview was conducted by visiting the participants’ homes. The purpose of the study was to understand why the participants did not visit the hospital for follow-up. Hence, interviewing them at their residence was identified to be a feasible and effective option.

### Study Duration

The study duration was 1 month, from January 15 to February 16, 2024.

### Participants

Participants were purposively selected for this research study. Participants who were part of a quasi-experimental study conducted to improve follow-up services in BEH and their service catchment area were chosen. Participants who had not attended even a single follow-up visit and participants who attended at least one follow-up were recruited for this qualitative study. Participants were selected irrespective of the intervention groups they were enrolled in during the quasi-experimental study. The participants were residing within a 20-km radius of BEH, and the interviews were conducted in the participant’s residence. Participants with relatively serious eye problems requiring multiple follow-ups were included. Based on the distance from the base hospital and the follow-up status, 65 participants were initially found eligible for the qualitative study. However, only 17 participants were available and consented. The interviews were conducted with these 17 participants.

### Data Collection Process

Topic guides were developed by an expert in qualitative research, who was our research guide, for the purpose of conducting in-depth interviews specifically for participants who had not attended even the first follow-up visit and those who attended at least one follow-up visit. Overall, 2 ophthalmic assistants, who were not a part of the main intervention study, conducted the interviews. These interviewers had a brief orientation on the topic guide and interview process to ensure consistency in the conduct of the interviews. The topic guides were piloted with a few participants before interviewing the actual participants. After providing prior information and obtaining written consent from each participant, the interview was carried out in their own communities, as it was not feasible for the participants to attend the hospital for interview purposes. During the interview process, one of the ophthalmic assistants conducted the interviews, and the other person was involved in observing and taking notes. Interviews were conducted with the parents or guardians of the children who received eye care services from BEH. The interview participants were the parents/service users who accompanied the child to the hospital during the initial checkup. All the interviews were audio-recorded using a smartphone with consent from participants. We chose to stop the interviews based on the principles of data saturation, that is, when no further information was provided by the participants interviewed.

### Data Analysis

We used the framework approach to analyze the qualitative data from this study. Interviews were audio-recorded, and the recorded interviews were transcribed verbatim. The interviews were conducted in the local language (Nepali). Hence, the audio recordings were first transcribed verbatim, and subsequently, the transcribed interview transcripts were translated and back-translated to ensure consistency in English by the other members of the study team. The transcribed data were read a multiple number of times to ensure familiarity. Codes were then identified to understand the framework for analysis. The codes and categories identified from the transcripts were then applied to that framework. The charted data were then carefully interpreted. We carried out member checking to triangulate data and to ensure data confirmability.

### Ethical Considerations

Ethics approval was obtained from the Ethical Review Board of the Nepal Health Research Council (ethical review board protocol registration number 761/2020 P and ClinicalTrials.gov number: NCT04837534). Written consent was obtained from the parents or guardians via a consent form before enrolling them in the study. All the information collected was secured and stored safely by the principal investigators. The data were completely anonymized for the purpose of privacy and confidentiality. The participants were not compensated for participating in the study.

## Results

### Overview

In total, 17 service users whose children were receiving services from BEH participated in the qualitative study. Details of the participants are provided in Table 1. Participants represented the actual communities in Nepal on important sociodemographics. We identified 4 key themes that provided invaluable information about the barriers and facilitators to follow-up as well as the experiences (positive or negative) of the participants that need to be considered in any future initiatives to improve follow-up in Nepal.

**Table 1.** Characteristics of study participants.

Interviewee	Relation with child	Age (years)	Education^a^	Profession	Child age (in years)	Approximate distance from hospital (in km)
1	Mother	25	Secondary	Housewife	2	5
2	Father	30	Secondary	Driver/business	6	5
3	Father	34	Secondary	Business	7	15
4	Mother	28	Primary	Housewife	2	7
5	Mother	35	Secondary	Business	9	18
6	Father	40	Secondary	Plumber	9	16
7	Mother	22	Illiterate	Housewife	1	54
8	Mother	40	Secondary	Housewife	10	34
9	Father	35	Secondary	Contractor	1	17
10	Father	30	Above secondary	Business	1	9
11	Mother	29	Secondary	Housewife	2	11
12	Mother	30	Secondary	Business	1	10
13	Mother	40	Illiterate	Housewife	4	12
14	Father	45	Secondary	Business	10	8
15	Father	40	Secondary	Unemployed	3	13
16	Mother	29	Secondary	Farming	4	6
17	Father	50	Primary	Retired/farming	10	8

a Illiterate: unable to read and write in Nepali; primary: up to grade 5; secondary: grades 6 to 10.

### Barriers to Follow-Up

The main reasons for barriers to follow-up were (1) ignorance about the eye problem and the potential solutions, (2) the poor financial situation of the service users, and (3) lack of satisfactory experience from the service providers during follow-up. Service users lacked awareness about the importance of regular follow-up for their children’s eye condition. Some also found it very uncomfortable to go out and discuss this with others in the community for further support. This was particularly because they were financially poor and could not afford to spend time and seek this advice as well as the treatment charges in hospitals that may be imposed when they follow advice from the community. Hence, they did not attend the follow-up visit as advised by the BEH follow-up intervention.

Many expressed financial constraints as a barrier to follow-up. The costs incurred in the treatment and travel were not affordable for them. Also, they were skeptical about whether the medications, treatment, and investigation would fall under the insurance subsidy or not. Therefore, it was difficult for the service users to abide by the advice related to follow-up that was suggested.

People keep asking us to take our child to different places but its only us who know our situation – Not easy

Many parents were busy and had to take off time from their routine schedules including their work commitments, which made it nonfeasible to comply with the advice for follow-up. As their children were studying and attending school, it was difficult to match their schedule to bring the child for follow-up as advised by the service providers. Lack of school attendance was considered by the service users as a huge loss. Also, the distance of the hospital from home was a barrier, as it cost time and money to travel to the hospital from their faraway residence.

My child needs to go to school, so difficult to come for follow-up during the school hours.

The child was not compliant with the treatment given, like not wearing the glasses prescribed. Some parents were unsure if the child was asked to bring it for follow-up or not. There were a few service users whose children felt better with the initial treatment and so did not continue to follow up.

I do not recall being called for follow up.

### Experiences and Perceptions

During the interview, service users felt that their presence was not given any importance. Service users expressed that BEH was the main center for eye care in Chitwan and it was mostly overcrowded, implying a long waiting time, not just at one counter but in all the counters, rooms, and departments they visited. The system and pathway that service users were advised to follow were perceived to be outdated, and there was no fast-track or exclusive service for those referred by their own staff and their own catchment area, thus making the experience not very satisfactory. This was especially the case for those who could not afford the service and for those who required access to the service on an urgent basis. The service users suggested that it would have been better if there was a fast-track eye checkup system, especially for very small children and those who were invited for a follow-up appointment.

One whole day was wasted for eye checkup at BEH, no one valued our time, we are not sure why did they ask us to come for making us wait for so long

The service users also felt that some hospital staff did not respond well. They expressed that the BEH support staff were busy on the phone and did not have a friendly approach. Some felt that there was miscommunication about the dates and time for follow-up and a lack of timely information provision and advice related to their felt needs. In addition, a few service users expressed that they were unable to recollect their experiences accurately as it has been a long time since they visited the hospital for follow-up.

I was there in the queue for 3 hours and when my turn came, I was handed out the paper and told to come the next day, that is unfair, they should have informed me on time, it is not possible for me to come here daily. I have a shop to run and so many errands at home!

Service users also shared their positive experiences during the follow-up. They expressed that BEH was well known in our communities; therefore, whenever there was any eye care need, service users preferred visiting BEH rather than other centers (medical or optical shops). Some service users liked that the system was available to access eye care and felt that their experience was comfortable at BEH. One the key features liked by service users was proper signage, which guided them in identifying and accessing different departments. The token system for registration and access to respective departments without any waiting time and smaller queues and the presence of a separate department for children, which made the experience comfortable for the kids and their parents.

It has a homely environment and feels like a second home.

Service users also felt that both clinical and nonclinical staff were well-behaved and had a friendly attitude. The doctors and other clinical staff used very simple and understandable advice. They expressed that the service providers had explained the procedures and treatment options clearly that made it very easy to understand and make decisions. Incidentally, some people experienced less crowd during their visit, so their experience was smooth and less time-consuming.

All staffs even the guard were very friendly to me.

Service users felt that the doctors provided good treatment, provided patient education, and stressed the importance of follow-up visits. The counselor and other clinical staff were observed to have stressed the role of proper treatment options and a good healthy lifestyle to reduce eye problems (such as reducing screen time, having a good healthy diet, and importance of outdoor activities for children).

The eye check-up went easily, it was not at all complicated as anticipated.

### Drivers for the Follow-Up

One of the important facilitators for implementing any intervention at BEH was the trustworthiness and brand name of the service provider. Being the oldest and biggest eye care center in Chitwan, BEH has earned its reputation; people perceive it to be the key service provider for that the region.

This is the oldest and biggest centre for eyes in Chitwan, why would I go anywhere else?

Service providers expressed that the eye camps were regularly held by the BEH at schools and localities, which refer patients to the base hospital (BEH). They felt that the service providers helped with information regarding the importance of preschool vision screening. The hospital also provided a national health insurance scheme, so it was very economical to get treatment there. There was also a subsidy system for low-income patients, so no one was denied basic eye care at BEH. Related to the intervention, participants felt that the reminder phone call from the hospital was very useful and helped people to remind themselves of the upcoming follow-up date.

## Discussion

### Principal Findings

This qualitative study aimed to explore the reasons for poor follow-up for eye care services and the experiences of service users receiving pediatric eye care at BEH. The findings revealed a complex interplay of factors influencing adherence to follow-up appointments, encompassing barriers, negative experiences, positive experiences, and facilitators. Lack of information and awareness about the eye problem and the required care, poor experience in accessing follow-up care, opportunity cost, and financial constraints have been the key reasons for poor follow-up. In addition, the COVID-19 pandemic and the naturally recovered service users who may not need a follow-up may be the contributors to poor follow-up that must be considered.

These reasons for poor follow-up were the commonly identified barriers to accessing any health or rehabilitation, particularly in the context of low- and middle-income countries [8]. In a country like Nepal, the geographical access barriers could be an additional drawback, which was not evident from this study [9-11]. However, the experiences of those who attended the follow-up provided very useful insights about the important aspects to ensure during the development of any pathway for follow-up [12,13]. They stress the importance of systematically developing a specific pathway that is sensitive to the context and culture of the service users in the catchment area [14-16]. They also highlight the need for sensitizing the service providers to the fact that the delivery of health care services may not have the same process and pathways for all service users [17].

The positive experiences from this study reveal some of the important aspects that need to be considered while developing such follow-up pathways for improving the effectiveness and quality of service provision [18]. This is applicable not just to eye care but to all kinds of health care provision services [19,20]. Service providers, including field workers and ground staff; organization of care; and delivery of services must be carefully optimized for improving service efficiency [21]. Our study highlights these important drivers for improving eye care services [22].

The implications of this study highlight the need for more research into the interventions for follow-up, especially for eye care services in Nepal and similar contexts. Much of the care that are globally available is provided within an institution [3-7]. However, for eye care services, this approach needs to be different, and it should include community-based care connecting service users in the community to the service providers at the institutions [23,24]. Technology can be a huge advantage in developing such kinds of interventions. Hence, more research is required on the systematic development and evaluation of comprehensive interventions that can promote a continuum of care [25].

This study also has a few practice and policy implications. Increasing awareness about the problem and potential solutions at the first point of contact can promote the information required to identify choices of care and make appropriate decisions about it. Similarly, the optimization of insurance coverage for socially disadvantaged communities and economically deprived service users by ratifying existing policies or expanding and implementing coverage to provide financial relief for service users can add value and improve service users’ experience positively [26].

### Limitations and Strengths

This study also comes with a few limitations. Since the initial experimental study was conducted during the COVID-19 pandemic, it was difficult to locate the participants for recruitment. We collected data 2 years post COVID-19, but soon after, the experimental study to test the intervention effectiveness was completed. Although this might appear to contaminate the results, the authors did not identify any potential limitations or recall issues related to the objective of this study during the study. Also, the other stakeholders, such as service providers and administrators, were not interviewed. Doing so might provide their perspectives for understanding the actual context. The interview was recorded in Nepali and was later translated in English. This may have resulted in inadequate reporting of the emotional aspects of the interview. These aspects need to be carefully considered in future studies on this topic. The study also had a few strengths. This study was actually an extension of a quasi-experimental study to understand the lack of statistically significant differences in the findings of that quantitative study. This study enabled us to understand the reasons for systematically developing and evaluating interventions. The participants were those with a need to attend follow-ups but did not turn up. Hence, the data from interviews relate very closely to the purpose of the study. Data triangulation, through repeated verification of audio recordings and transcriptions, was completed. We also carried out member checking and added rigor to this study.

### Conclusion

This study provides immensely valuable insights. It highlights the need for systematic development of interventions to address the unmet need for eye care services in the community through innovative, scalable solutions. As a next step, the BEH team will be working to develop such scalable solutions for Nepal. Such interventions will also need to be optimized for similar settings and countries to meet the goals of universal health coverage, vision 2030, and sustainable development goals worldwide.

## Abbreviations

BEH: Bharatpur Eye Hospital
RECON: Retina Eye Care of Nepal

## References

[1] Gurung R, Oli RU (2021). Primary eye care in Nepal: current situation and recommendations for integration. Community Eye Health.

[2] Shrestha A, Shrestha C, Karki P, Gurung HM, Naito T (2020). Strengthening retina eye care services in Nepal: retina eye care of Nepal project. BMC Health Serv Res.

[3] Shrestha R, Singh P, Dhakwa P (2023). Augmenting the referral pathway for retinal services among diabetic patients at Reiyukai Eiko Masunaga Eye Hospital, Nepal: a non-randomized, pre-post intervention study. BMC Health Serv Res.

[4] Rai SKC, Thapa H, Kandel RP, Ishaq M, Bassett K (2014). Clinical and cost impact of a pediatric cataract follow-up program in western Nepal and adjacent northern Indian States. J AAPOS.

[5] Bhattarai B, Thapa HB, Bashyal S (2024). Structured counselling and regular telephonic follow up to improve referral flow and compliance in Nepal for Diabetic Retinopathy(SCREEN-D Study): a randomised controlled trial. BMC Health Serv Res.

[6] Lin H, Chen W, Luo L (2012). Effectiveness of a short message reminder in increasing compliance with pediatric cataract treatment: a randomized trial. Ophthalmology.

[7] Shrestha M, Bhandari G, Kamalakannan S (2023). Evaluating the effectiveness of interventions to improve the follow-up rate for children with visual disabilities in an eye hospital in Nepal: nonrandomized study. JMIR Pediatr Parent.

[8] Ibbotson JL, Luitel B, Adhikari B (2021). Overcoming barriers to accessing surgery and rehabilitation in low and middle-income countries: an innovative model of patient navigation in Nepal. World J Surg.

[9] Syed ST, Gerber BS, Sharp LK (2013). Traveling towards disease: transportation barriers to health care access. J Community Health.

[10] Gogate P, Patil S, Kulkarni A (2014). Barriers to follow-up for pediatric cataract surgery in Maharashtra, India: how regular follow-up is important for good outcome. The Miraj Pediatric Cataract Study II. Indian J Ophthalmol.

[11] Eriksen JR, Bronsard A, Mosha M, Carmichael D, Hall A, Courtright P (2006). Predictors of poor follow-up in children that had cataract surgery. Ophthalmic Epidemiol.

[12] Thompson AC, Thompson MO, Young DL (2015). Barriers to follow-up and strategies to improve adherence to appointments for care of chronic eye diseases. Invest Ophthalmol Vis Sci.

[13] Bergin RJ, Yao-Dong Yu A, White V, Emery J (2024). Discussions with patients about referral pathways and costs in the diagnosis and treatment of colorectal cancer in Victoria, Australia. Aust J Gen Pract.

[14] Spinelli M, Preti E, Kassa TT (2023). Cultural considerations in the assessment of sensitivity in low-income caregivers in Ethiopia. Front Psychol.

[15] Brilliant GE, Lepkowski JM, Zurita B, Thulasiraj RD (1991). Social determinants of cataract surgery utilization in south India. The Operations Research Group. Arch Ophthalmol.

[16] Snellingen T, Shrestha BR, Gharti MP, Shrestha JK, Upadhyay MP, Pokhrel RP (1998). Socioeconomic barriers to cataract surgery in Nepal: the South Asian cataract management study. Br J Ophthalmol.

[17] Røsstad T, Garåsen H, Steinsbekk A, Sletvold O, Grimsmo A (2013). Development of a patient-centred care pathway across healthcare providers: a qualitative study. BMC Health Serv Res.

[18] Seys D, Bruyneel L, Deneckere S (2017). Better organized care via care pathways: a multicenter study. PLoS One.

[19] Rai V (2023). COVID-19 and kidney: the importance of follow-up and long-term screening. Life (Basel).

[20] Papageorgiou C, Apalla Z, Manoli SM, Lallas K, Vakirlis E, Lallas A (2021). Melanoma: staging and follow-up. Dermatol Pract Concept.

[21] Yinusa A, Faezipour M (2023). Optimizing healthcare delivery: a model for staffing, patient assignment, and resource allocation. Appl Syst Innov.

[22] Singh P, Jain N, Verma S, Sharma B (2023). Commentary: ophthalmology training programs: optimization of human resource to supplement clinical expertise and strengthen eye care delivery systems. Indian J Ophthalmol.

[23] Bechange S, Buttan S (2022). Effectiveness of community-based eye care: process and considerations. Lancet Glob Health.

[24] Musch DC, Andrews C, Schumann R, Baker J (2020). A community-based effort to increase the rate of follow-up eye examinations of school-age children who fail vision screening: a randomized clinical trial. J AAPOS.

[25] Das T, Dhoj Sapkota Y (2021). Promoting technology-enabled primary eye care in South-East Asia. Community Eye Health.

[26] Ghimire S, Ghimire S, Khanal P, Sagtani RA, Paudel S (2023). Factors affecting health insurance utilization among insured population: evidence from health insurance program of Bhaktapur district of Nepal. BMC Health Serv Res.

